# Supplementary material for: Cellular Mechanosensitivity: Validation of an Adaptable 3D-Printed Device for Microindentation
Source: Nanomaterials (Basel). 2022 Aug 5;12(15):2691. doi: 10.3390/nano12152691 (PMC9370482; doi:10.3390/nano12152691)
Supplement: Supplementary file 1 [file nanomaterials-12-02691-s001.zip › nanomaterials-1811873-Supplementary Materials.pdf]

Supplementary Materials

Cellular Mechanosensitivity: Validation of an Adaptable 3D-Printed Device for Microindentation

Giulio Capponi [1,2,*], Martina Zambito [2], Igor Neri [1], Francesco Cottone [1], Maurizio Mattarelli [1], Massimo Vassalli [3,*], Silvia Caponi [4] and Tullio Florio [2,5,*]

[1] Dipartimento di Fisica e Geologia, Università di Perugia, I-06100 Perugia, Italy; igor.neri@unipg.it (I.N.); francesco.cottone@unipg.it (F.C.); maurizio.mattarelli@unipg.it (M.M.)
[2] Sezione di Farmacologia, Dipartimento di Medicina Interna, Università di Genova, 16132 Genova, Italy; zambito.martina@yahoo.com
[3] James Watt School of Engineering, University of Glasgow, Glasgow G12 8LT, UK; massimo.vassalli@glasgow.ac.uk
[4] Istituto Officina dei Materiali, Italian National Research Council (IOM-CNR), Unit of Perugia, c/o Department of Physics and Geology, University of Perugia, Via A. Pascoli, I-06123 Perugia, Italy; silvia.caponi@cnr.it
[5] Istituto di Ricovero e Cura a Carattere Scientifico, Ospedale Policlinico San Martino, 16132 Genova, Italy
* Correspondence: giulio.capponi@studenti.unipg.it (G.C.); massimo.vassalli@glasgow.ac.uk (M.V.); tullio.florio@unige.it (T.F.)

Human-Piezo1 expression

In order to characterize the Piezo1 expression in A1 cells, upon the transfection with the human-Piezo1-GFP plasmid (A1–Piezo1), real time polymerase chain reaction (PCR) was performed in the GFP positive clones and confirmed the presence of human-Piezo1 gene (Figure S1).

Both mouse and human Piezo1 primers were selected and normalized to the expression of internal controls genes. The relative expression of human Piezo1 grows, as expected, in A1-Piezo1, with a slight not significant reduction in mouse Piezo1 expression.

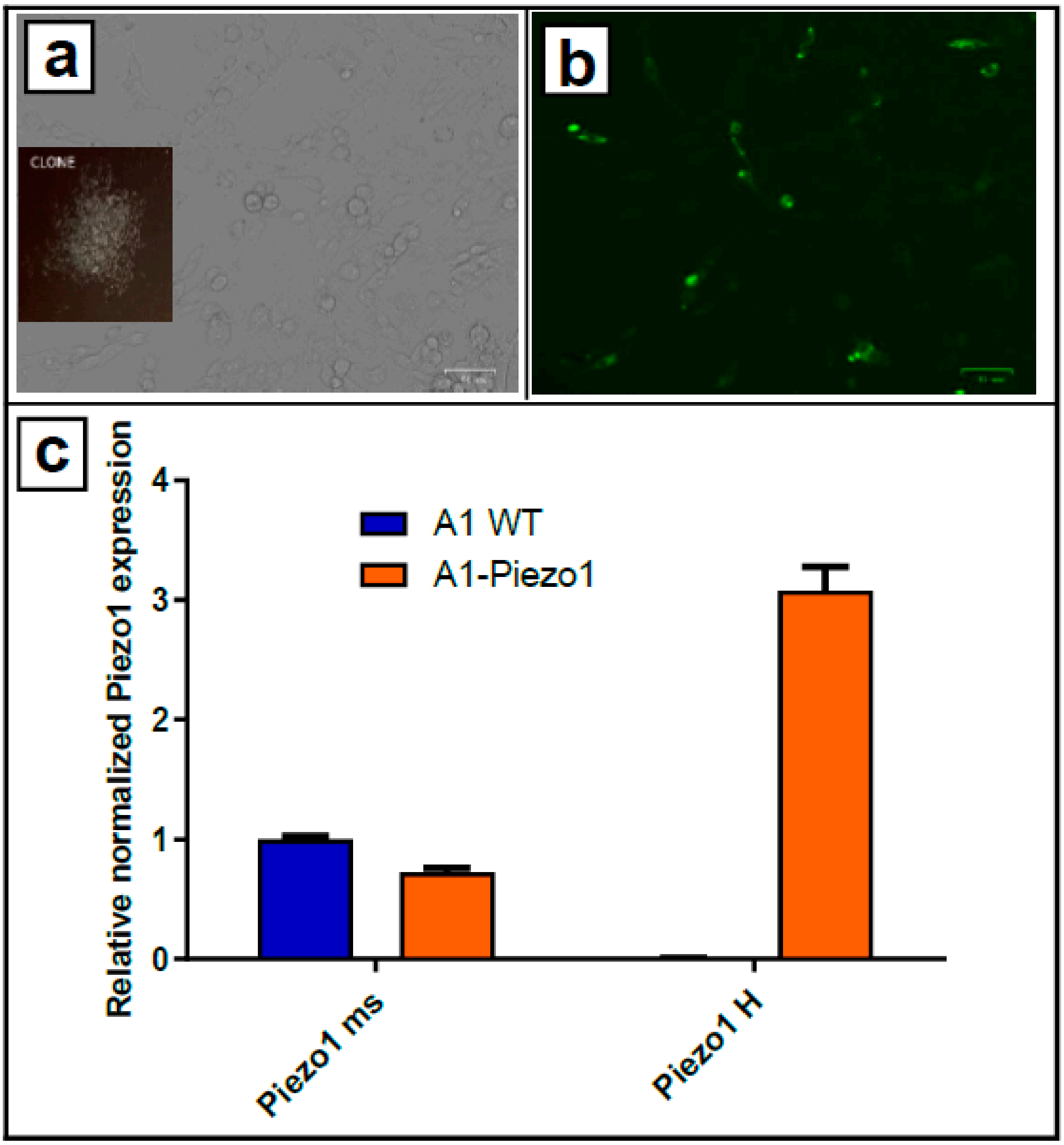

Figure S1. Human-Piezo1-GFP selection and real time PCR of A1. A1-Piezo1 A1 Piezo1 cells were selected from a first antibiotic selection and a second selection based on the GFP fluorescence of the Piezo1 plasmid. The image shows one of the selected GFP positive clones in phase contrast **(a)** and the corresponding GFP fluorescence **(b)** Furthermore, the transfection with human-GFP-Piezo1 plasmid was verified by real time PCR: the diagram **(c)** expresses the relative level of Piezo1 mouse (Piezo1 ms) and human-Piezo1-GFP (Piezo1 H) mRNA analyzed compared to internal standards 28 GAPDH and H3.

Minimum vertical displacement of the linear translation stage 31

The translation stage (see Section 2.5 and Figure 1, a) presents an expected minimum displacement of ~24.4 nm. The latter value results from the mechanical reduction (1:20480) of the actuating screw pitch (0.5 mm). The total reduction is the combination of the reduction from the lever system (1:5), the reduction due to the gear jointed to the step motor (1:2) and the internal reduction of the step motor in full-step configuration (1:2048) [1].

The actual minimum displacement of the stage was measured by interferometric acquisition through the calibration set-up mentioned in Section 3.2. The stage was driven through a sequence of 1 μm displacements both in positive and negative Z axis direction. The assessment of actual and target positions consents to obtain a correction factor for the minimum displacement, resulting in ~21.1 nm and ~18.9 nm (Figure S2).

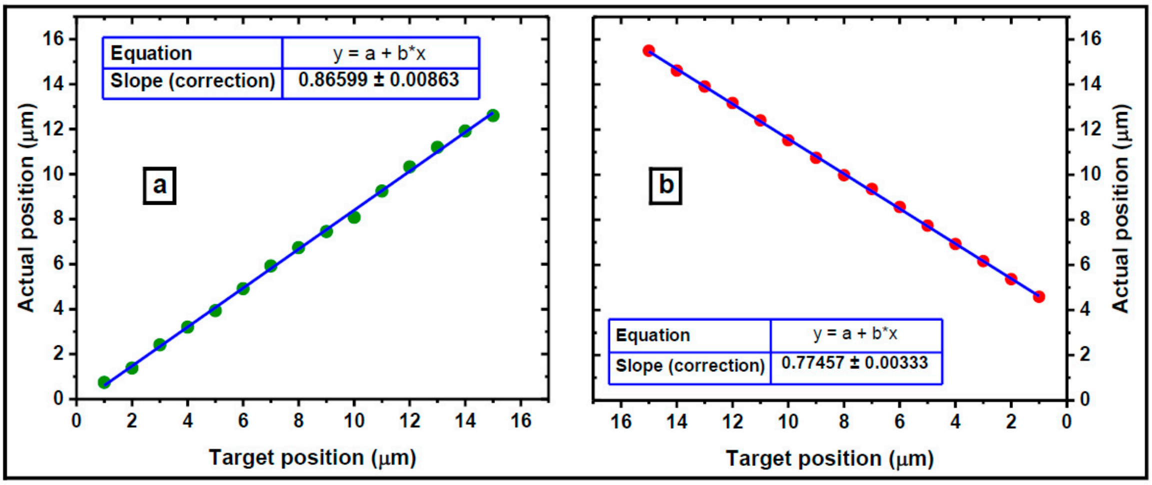

Figure S2. Minimal vertical displacement calibration of the motion stage. A set of 15 displacements with nominal movements of 1 μm have been exerted to positive (**a**) and negative (**b**) direction. A correction factor was evaluated as the slope of the linear fit between the actual and the target positions reached by the retroreflector. Product between the correction factor and the nominal step displacement (~24.4 nm) returns the actual minimum displacement: ~21.1 nm and ~18.9 nm for positive and negative direction respectively.

Cell disruption vs. Indentation depth 50

Both responsive and unresponsive cells were indented, by increasing probe vertical displacements D (see Section 2.7), until the rupture of the membrane occurred at an indentation depth δ_R. The distribution of δ_R, for A1 WT and A1-Piezo1 cells is reported in Figure S3. It is worth noticing that the mean of δ_R results ≃2 μm greater than the indentation depth δ for activation (Figure 6, c).

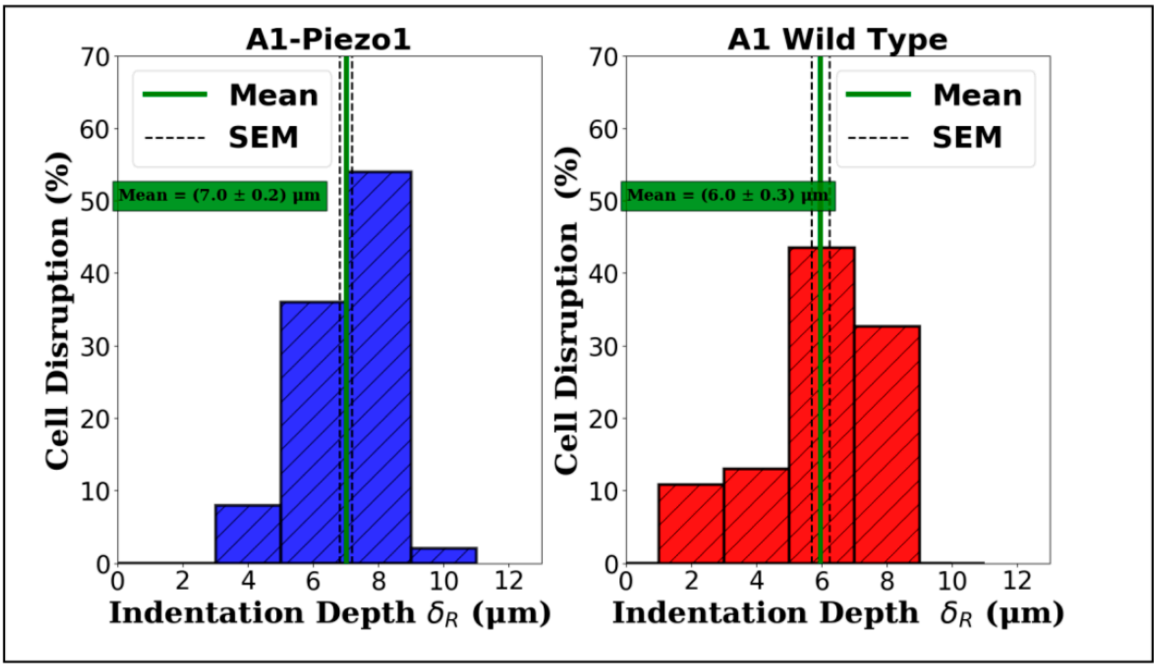

Figure S3. Indentation depth for cellular disruption. Rate of cell disruption events at a given rupture indentation depth δ_R.

References

[1] Q. Meng, K. Harrington, J. Stirling, and R. Bowman, 'The OpenFlexure Block Stage: sub-100 nm fibre alignment with a monolithic plastic flexure stage', *Opt. Express*, vol. 28, no. 4, p. 4763, 2020, doi: 10.1364/oe.384207.
